# Dealing with mold infestation in hospitals and medical practices

**DOI:** 10.3205/dgkh000594

**Published:** 2025-11-21

**Authors:** Julia Hurraß, Eva-Brigitta Kruse-Wehner, Steffen Engelhart, Caroline E.W. Herr, Ludger Klimek, Dennis Nowak, Jörg Steinmann, Jens-Oliver Steiß, Janine Zweigner, Gerhard A. Wiesmüller

**Affiliations:** 1 HYGIUM – Center for Hygiene and Environmental Medicine, Cologne, Germany; 2 Institute for Translational Research, CECAD Cluster of Excellence, University of Cologne, Cologne, Germany; 3 Laboratory Dr. Wisplinghoff, Cologne, Germany; 4 Institute for Hygiene and Public Health, University Hospital Bonn, Germany; 5 Bavarian State Office for Health and Food Safety Munich, Germany; 6 Environmental Health Protection and Prevention Working Group, Institute and Polyclinic for Occupational, Social and Environmental Medicine, Member of the German Center for Lung Research, Munich University Hospital, Munich, Germany; 7 Center for Rhinology and Allergology, Wiesbaden, Germany; 8 Institute and Clinic for Occupational, Social and Environmental Medicine, Member of the German Center for Lung Research, DZL, CPC (Comprehensive Pneumology Center Munich), LMU Munich University Hospital, Germany; 9 Institute for Clinical Hygiene, Medical Microbiology and Clinical Infectiology, Paracelsus Medical University Nuremberg Hospital, Germany; 10 Center for Pediatrics and Adolescent Medicine, University Hospital Gießen and Marburg GmbH, Giessen, Germany; 11 Specialist practice for allergology and pediatric pneumology Fulda, Germany; 12 Central Hospital Hygiene, University Hospital Cologne, Germany; 13 Institute for Occupational, Social and Environmental Medicine, RWTH Aachen University Hospital, Germany

**Keywords:** moisture, mold, hospital, dental practice, medical practice, infection control, hygiene

## Abstract

**Introduction:**

Mold, which includes the components and metabolic products of mold fungi as well as other factors associated with moisture, such as yeasts, bacteria (actinobacteria) and mites, occurs indoors when there is increased moisture. In addition to known causes of damp/mold damage such as structural defects (including thermal bridges, rising damp, insufficient ventilation), water damage and incorrect ventilation and heating options, construction work plays an important role in hospitals and medical practices.

**Methods:**

The AWMF S2k guideline “Medical clinical diagnostics for indoor mold exposure” was first published in 2016. Following a new systematic literature search, an updated version of this guideline was published in October 2023 with the cooperation of various medical disciplines and other experts. The guideline is intended to close the existing knowledge gap in terms of rational and efficient medical diagnosis in cases of indoor mold contamination. Recommendations are given on general and specific examination methods. Non-evidence-based diagnostic methods, some of which are still used in practice, are not recommended. Furthermore, treatment options for health problems and diseases caused by mold fungi are presented. It is explained why, in most cases, indoor measurements of molds, their components or metabolites are not required for medical evaluation.

**Results:**

Individuals who are immunosuppressed (grade 2 and 3 of the immunosuppression grades according the Commission for Hospital Hygiene and Infection Prevention [KRINKO] at the Robert Koch Institute [RKI]), or are suffering from severe influenza, severe COVID-19, cystic fibrosis and uncontrolled bronchial asthma are at increased risk of exposure to mold fungi. For these patients, exposure to mold damage must be ruled out in the clinic and practice as well as in the home environment.

**Conclusion:**

If, despite all precautionary measures, moisture/mold damage can be detected in the environment of these particularly vulnerable patients, it must be remediated immediately. Indoor measurements of mold exposure are not indicated for medical reasons. Rather, all measures that help to avoid moisture damage are crucial for prevention.

## Zusammenfassung

**Einleitung:**

Schimmel, worunter die Bestandteile und Stoffwechselprodukte von Schimmelpilzen, aber auch weitere mit Feuchtigkeit assoziierte Faktoren wie Hefen, Bakterien (Aktinobakterien) und Milben subsumiert werden, tritt in Innenräumen bei erhöhter Feuchtigkeit auf. Neben bekannten Ursachen für Feuchte-/Schimmelschäden wie baulichen Mängeln (u.a. Wärmebrücken, aufsteigende Feuchtigkeit, unzureichende Lüftungsmöglichkeiten), Wasserschäden sowie fehlerhaftem Lüftungs- und Heizverhalten spielen in Krankenhäusern und Praxen Baumaßnahmen eine wichtige Rolle.

**Methoden:**

Im Jahr 2016 wurde die AWMF-S2k-Leitlinie „Medizinisch klinische Diagnostik bei Schimmelpilzexposition in Innenräumen“ erstmals veröffentlicht. Nach einer erneuten systematischen Literaturrecherche wurde unter Einbeziehung verschiedener medizinischer Fachrichtungen und weiterer Experten/Expertinnen im Oktober 2023 eine aktualisierte Fassung dieser Leitlinie herausgegeben. Die Leitlinie soll die bestehende Wissenslücke für eine rationale und effiziente medizinische Diagnostik bei Schimmelpilzbelastungen in Innenräumen schließen. Es werden Empfehlungen zu allgemeinen und speziellen Untersuchungsmethoden gegeben. Dabei wird von nicht-evidenzbasierten diagnostischen Methoden, die teilweise immer noch in der Praxis Anwendung finden, abgeraten. Weiterhin werden Therapieoptionen für durch Schimmelpilze verursachte Gesundheitsprobleme und Erkrankungen dargestellt. Es wird erläutert, warum für die medizinische Bewertung in den meisten Fällen keine Innenraummessungen von Schimmelpilzen, ihren Bestandteilen oder Metaboliten erforderlich sind.

**Ergebnisse:**

Personen unter Immunsuppression (Grad 2 und 3 der Immunsuppressionsgrade gemäß der Kommission für Krankenhaushygiene und Infektionsprävention [KRINKO]), mit schwer verlaufender Influenza, mit schwer verlaufender COVID-19, mit Mukoviszidose (zystischer Fibrose) und mit einem unkontrollierten Asthma bronchiale weisen eine erhöhte Gesundheitsgefährdung gegenüber Schimmelpilzexpositionen auf. Für diese Patienten/Patientinnen muss in Klinik und Praxis sowie im häuslichen Umfeld eine Exposition gegenüber Schimmel-/Feuchteschäden ausgeschlossen werden.

**Schlussfolgerungen:**

Sollte trotz aller Vorsichtsmaßnahmen im Umfeld dieser besonders gefährdeten Patienten/Patientinnen ein Schimmel-/Feuchteschaden nachweisbar sein, muss dieser unmittelbar saniert werden. Aus medizinischer Indikation sind Innenraummessungen einer Schimmelpilzexposition nicht indiziert. Vielmehr sind zur Prävention alle Maßnahmen entscheidend, die dazu beitragen, Feuchteschäden zu vermeiden.

## Introduction

Indoor mold growth is always related to increased humidity [1], [2], [3]. In a study of dampness and mold in 7,127 homes in 22 centers across Europe (including Germany) with on-site inspections in 3,118 homes, the frequency of self-reported water damage (10%), damp patches (21%) and mold (16%) was comparable to observed dampness (19%) and observed mold (14%) [4]. The proportion of homes in Germany with visible mold infestation ranges from 5–20% [5], [6], [7], [8], [9]. 

Moisture/mold damage also occurs in hospitals [10], [11], [12], [13], [14], [15], [16], [17], [18], [19], [20], [21], [22], [23] and medical practices [24], [25].

In addition to the usual causes of moisture/mold damage, e.g., structural defects (including thermal bridges, rising damp, insufficient ventilation), water damage or usage behavior (incorrect ventilation and heating), mold exposure in hospitals and medical practices is often associated with construction work.

The “Guidelines for the prevention, detection and remediation of mold infestation in buildings” published by the Federal Environment Agency [26] are available for the detection, assessment, search for causes and remediation of mold growth indoors. A good instrument for remediation control is provided by WTA data sheet 4–12 [27], which includes total spore measurements in accordance with DIN ISO 16000-20 [28] after mobilization of existing dust deposits to check the success of cleaning.

For health assessment of indoor mold exposures, the following sources are available: a statement by the RKI Commission “Methods and Quality Assurance in Environmental Medicine” [29], a WHO guideline [3], answers to frequently asked questions on health risk assessment of indoor mold exposures by the Bioaerosols Working Group of the German Society for Hygiene, Environmental Medicine and Preventive Medicine (GHUP) [30], [31], [32], [33], [34], [35], [36], [37], [38], [39], [40], and the AWMF mold guideline “Medical clinical diagnostics for indoor mold exposure“ [41], which was created in 2016 and updated in 2023 [1], [2], AWMF register no. 161/001.

None of these publications are address moisture/mold damage in hospitals and medical practices; some even explicitly exclude it. This article aims to close this gap.

## General health risk assessment of indoor exposure to moisture/mold

Epidemiological studies and case reports (e.g., [42]) consistently show a connection between indoor damp/mold damage and health effects, especially respiratory complaints, eye, nose and throat irritation, nasal congestion, wheezing, dry cough, sleep disturbance, snoring and fatigue [43], [44]. The AWMF guideline [1], [2] is essentially limited to clinical presentations rather than symptoms. The evidence for associations between moisture/mold damage and the different health effects is shown in Table 1 (Tab. 1).

Whether mold poses a health risk depends largely on the disposition of the exposed persons.

### Persons at risk

Persons at risk, requiring special protection:

1. Persons with immunosuppression according to the classification of the Commission for Hospital Hygiene and Infection Prevention (KRINKO) at the Robert Koch Institute (RKI) [45]
2. Persons with severe influenza
3. Persons with severe COVID-19
4. Persons with cystic fibrosis (mucoviscidosis)
5. Persons with bronchial asthma

Causality between specific mold exposure and specific health complaints and symptoms cannot be indubitably established in individual cases.

A clear cause-and-effect relationship cannot be established from the simple correspondence between measured mold exposure and possible health effects. The reasons for this are explained in more detail, using the possible health effects of indoor mold exposure listed below.

### Infections

**Figure 1:**
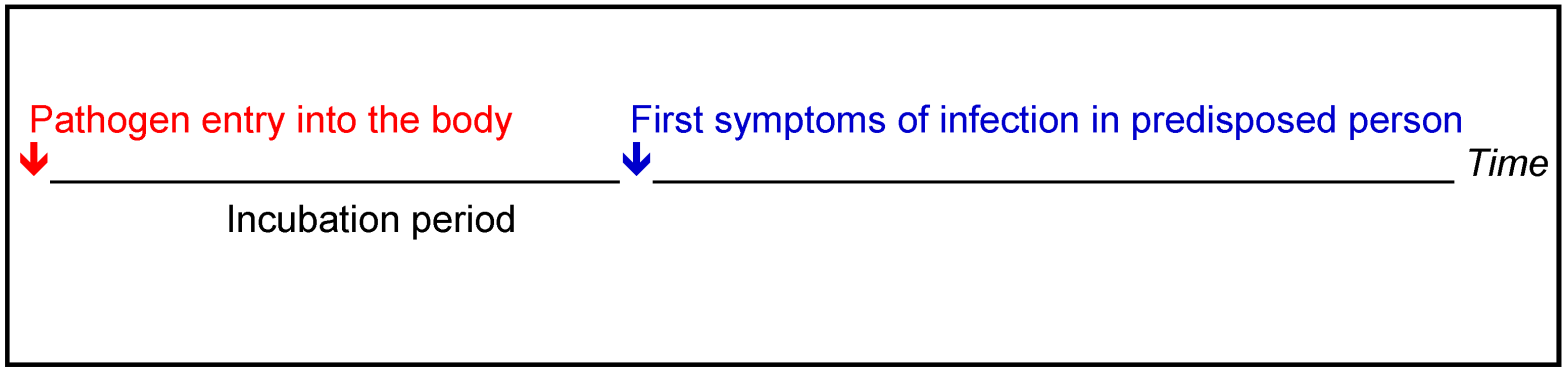
Incubation period [46], [47]

Every infectious disease is preceded by an incubation period. This is the period between the entry of an infectious agent into the human body and the appearance of the first symptoms of infection, i.e., the outbreak of the infectious disease (Figure 1 (Fig. 1)). For example, an incubation period of days to weeks is specified for invasive aspergillosis [46], [47].

Since molds are ubiquitous, the cause of a mold infection cannot be reliably identified on the basis of indoor exposure to one or more molds with a risk of infection according to the German Ordinance on Biological Agents as postulated by experts [48]. The mold fungus may have entered the human body from a different environment, such as the outside air, a compost pile, the organic-waste garbage can, another indoor room, etc. In contrast to bacterial pathogens, it is rarely possible to detect indistinguishability between patient isolates and environmental isolates of molds using molecular biological methods. Only very few ubiquitous molds can be considered infectious agents in humans (e.g., *Aspergillus (A.) fumigatus*).

A prerequisite for a mold infection in humans is a pronounced weakness of the immune system (immunosuppression). This disease susceptibility (disposition) can only be assessed by a physician based on the three risk groups of the Commission for Hospital Hygiene and Infection Prevention (KRINKO) at the Robert Koch Institute [45].

If there is such a disposition (immunosuppression) and a source of mold indoors or in the outside air, exposure must be stopped immediately. Affected patients must be informed by their doctor about the necessary measures to avoid exposure. A metrological objectification of mold exposure to one or more molds with risk of infection according to the Biological Agents Ordinance has no benefit for the immediate protection of the immunocompromised person, but in fact carries the risk of a potentially life-threatening extension of exposure [46], [47].

### Sensitizations and allergies

**Figure 2:**
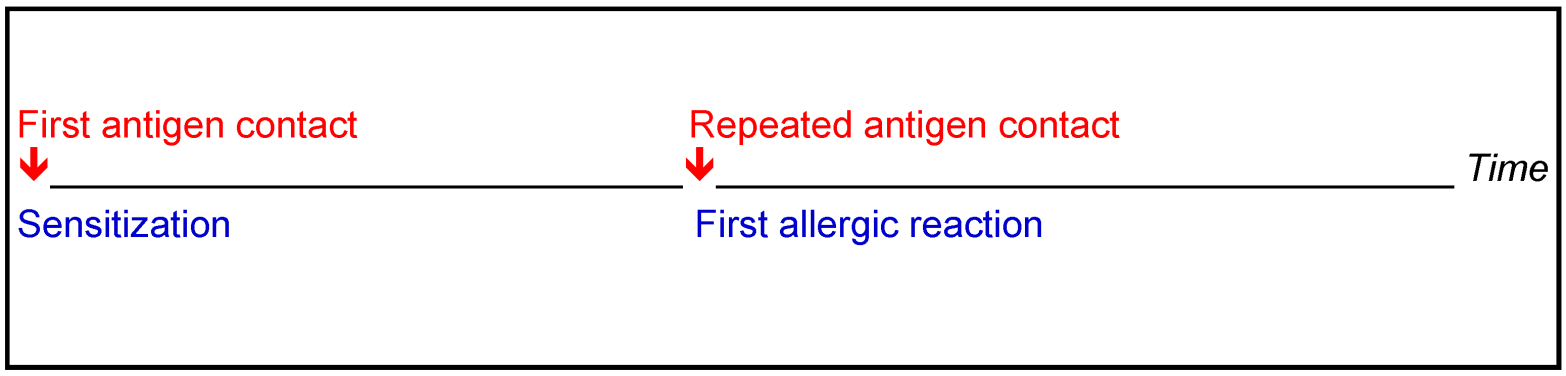
Temporal relationship between sensitization and first allergic reaction [46], [47]

Sensitization is a misdirected specific immune reaction following initial contact with an antigen and the formation of antibodies (IgE). Only after renewed contact with the same trigger are various mediators released that cause a clinical "allergic" reaction. Sensitization is not synonymous with allergic symptoms and does not always lead to an allergy (Figure 2 (Fig. 2)). The time interval between sensitization and the first allergic reaction varies [46], [47]. 

Differentiation between sensitization and allergy after exposure to mold fungi is not possible by indoor measurements (CFU/m^3^) [49].

People who are already sensitized to mold fungi may experience allergic reactions due to moisture/mold damage indoors. In this case, too, no measurement-based objectification is required, but rather the cessation of exposure. This is particularly important for patients with allergic asthma, as they may react with an asthma attack.

Since no (valid) test extracts for the detection of sensitization, so-called allergy tests, are commercially available for most indoor-associated molds, test results for the detection of a mold allergy cannot be used for a risk assessment, nor can the results of mold measurements in rooms used by the persons concerned.

The most important means of preventing sensitization and allergic reactions if sensitization already exists is to stop exposure, i.e., the mold infestation must be remediated [46], [47].

### Toxic effects

There is relatively long latency period between the intake of a potentially toxic substance and possible health effects. 

**Figure 3:**
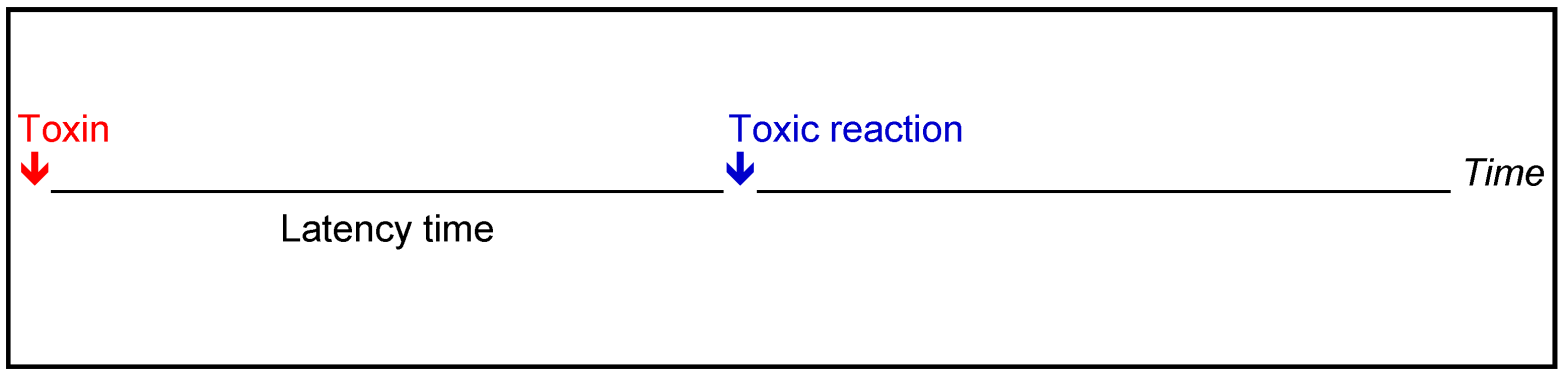
The latency time [46], [47]

This refers to the period between the penetration of a toxin into the human body and the appearance of the first symptoms of poisoning (Figure 3 (Fig. 3)) [46], [47].

Exposure to moisture/mold damage is complex and variable. In addition to spores, cell fragments, metabolic products and mycotoxins from mold fungi, bacteria (including actinobacteria), MVOC (microbial volatile organic compounds produced by mold fungi), β-glucans, mannans, ergosterol, endotoxins, bioaerosol allergens, and mite allergens are also present. Because a quantitative health risk assessment for individual components of the bioaerosol is not possible, it is not necessary to determine individual components regarding a toxic effect when assessing a mold infestation.

It is known that in cases of moisture damage, some molds produce mycotoxins as secondary metabolites that are bound to spores, mycelium and cell fragments as components of house dust and bioaerosols, contributing to exposure and presumably to inflammatory/irritative mucosal reactions.

The concentrations of mycotoxins measured indoors to date for mold infestation are very low, so that acute toxic effects (mycotoxicosis) are not to be expected [50].

However, it has not yet been clarified in detail which other health effects and consequences may be attributed to this. Both antagonistic and synergistic effects have been described in the interaction of the diverse components of the bioaerosol of mold fungi and bacteria. In cases of massive moisture damage, the formation of mycotoxins and endotoxins (components of bacteria) must potentially be expected [51]. An increased release of mycotoxins can occur with such dried damage, especially during remediation measures, if large quantities of spores and dust are mobilized. In these cases, strict attention must be paid to the relevant occupational safety measures [52].

Determining mycotoxins is irrelevant for the medical assessment of moisture/mold damage. There is currently no indication for determining mycotoxins in blood or urine in medical diagnostics for people with indoor mold exposure. The routine determination of mycotoxins in indoor air or in mold-contaminated building materials has neither medical diagnostic significance nor is it necessary for a remediation decision, as any relevant mold infestation must be removed immediately, regardless of whether mycotoxins have been formed or not [46], [47].

As a matter of principle with all chronic illnesses, it must be ensured that the living conditions are hygienically impeccable, even without evidence or with insufficient proof of a connection to moisture damage and/or mold exposure. If there are hygienic indications or anamnestic evidence of moisture damage and/or mold exposure, the primary causes must always be eliminated, as with all moisture damage.

## Risk analysis and risk assessment

### Risk of infection

The risk of infection from mold species regularly found indoors is low for healthy people. Most species are classified in risk group 1 and a few in 2 (*A. fumigatus, A. flavus*) of the Biological Agents Ordinance [53].

For occupational activities (handling) with mold fungi, the current Biological Agents Ordinance applies, according to which the infection risks of biological agents are divided into four risk groups [54], with mold fungi being divided into the risk groups 1 and 2.

- **Risk group 1:** Biological agents that are unlikely to cause disease in humans.
- **Risk group 2:** Biological agents that can cause disease in humans and may pose a risk to workers; the substance is unlikely to spread to the population; effective prevention or treatment is usually possible.
- **Risk group 3:** Biological agents that may cause serious disease in humans and pose a serious risk to workers; there may be a risk of spreading to the population, but effective prevention or treatment is usually possible.
- **Risk group 4:** Biological agents that cause serious disease in humans and pose a serious risk to workers; the risk of spreading to the population may be high; effective prevention or treatment is not normally possible (risk group 4 does not include fungi).

Mold mycoses are opportunistic infections, requiring the presence of a compromised immune system in exposed persons. Thermotolerant mold species of risk group 2 (e.g., *A. fumigatus, A. terreus, A. niger, A. flavus, Emericella nidulan*s or mesophilic *Fusarium* spp.) of the “TRBA 460: Classification of molds into risk groups” [53] of the Biological Agents Ordinance [54] rarely cause infections in healthy, immunocompetent persons, but can trigger invasive mycoses in people whose immune system is suppressed due to illness or other circumstances [55]. The WHO offers a similar assessment in its current “WHO fungal priority pathogens list to guide research, development and public health action” [56].

According to the recommendation of the Commission for Hospital Hygiene and Infection Prevention (KRINKO) at the Robert Koch Institute [45], immunosuppressed persons can be divided into three risk groups (Table 2 (Tab. 2)):

1. Moderate immunosuppression/deficiency
2. Severe immunosuppression/deficiency
3. Very severe immunosuppression/deficiency

Particularly at risk are (list with decreasing risk) patients with tumor disease, especially with underlying hemato-oncological disease (e.g., leukemia, lymphoma), acute myelogenous leukemia (AML), acute lymphocytic leukemia (ALL), allogeneic stem cell transplantation, autologous stem cell transplantation, solid organ transplantation, HIV infection, other immunosuppression (e.g., prolonged high-dose glucocorticoid therapy), aplastic anemia, cystic fibrosis and many others [57], [58], [59], [60], [61], [62]. Acute myelogenous leukemia (AML) is associated with the highest incidence of invasive mold infections (about 12%) and most mold infections (about 8%). This is followed by acute lymphocytic leukemia (about 4%). Among the procedures, allogeneic hematopoietic stem-cell transplantation (alloSZT) is associated with a very high incidence of mold infections [57].

In addition, critically ill patients in intensive care units are at risk of *Aspergillus* infection [63], [64], [65], [66]. Patients with influenza [67], [68], [69], [70], [71], [72] and patients with COVID-19 [67], [73], [74], [75], [76], [77], [78], [79], [80] also have an increased risk of contracting fungal infections. The same applies to other severe respiratory viral infections [81], [82].

Due to the steady increase in the proportion of immunocompromised individuals in the population and the ever-longer lifespan of these people, it cannot be ruled out at present that mold infections may become an increasing risk factor for the health of this population group [45].

A numerical risk cannot be derived based on the current state of knowledge; only a semi-quantitative risk assessment is possible. Risk matrix 1 (Table 3 (Tab. 3)) shows a semi-quantitative risk assessment of the risk of infection from indoor molds. 

### Sensitization/allergy risk

In principle, sensitization and triggering of a clinically symptomatic allergy in healthy people after inhalation of spores and other mold components (e.g., mycelium) is possible. Compared to other environmental allergens, such as allergens from fur-bearing pets, grass and tree pollen or house dust mites (approx. 15 to 30% [83], [84]), the sensitizing potential of molds is estimated to be significantly lower [85], [86], [87], [88]. Both population-based and patient-based studies show a comparatively low sensitization prevalence of 3–22.5% across Europe [89], [90], [91], which varies greatly depending on the type of mold and shows a north-south gradient (low sensitization prevalence in Finland, relatively high in Greece) [91].

It is generally assumed that there are over one million mold species. To date, around 350 mold species have been listed as potentially sensitizing (https://www.allergome.org/). However, it cannot be concluded from this information how high the proportion of sensitizing mold species is overall. The criteria of the World Health Organization and International Union of Immunological Societies (WHO/IUIS) for classifying an allergen are currently met by 107 mold proteins from 43 mold species (https://www.allergome.org/). Only a few molds are available as test allergen solutions, and typical indoor fungal allergen extracts are largely lacking [92], [93].

From an allergological point of view, the dose dependence of exposure (in colony-forming units [cfu]) is not the only decisive factor for the clinical reaction after a patient has been sensitized to mold fungi. Sensitization with the formation of specific IgE antibodies and the triggering of allergic reactions occurs at the level of proteins or peptide components. It is therefore not necessary that whole spores or intact mold mycelium be present. Rather, the allergenicity depends on the proteins or peptides as well as the susceptibility of the exposed person, so that an antigen becomes an allergen and sensitization, or, given repeated contact, an allergy can result. This is because an antigen only becomes an allergen when the antigen reacts with a person’s immune system, which responds with an IgE reaction. According to the WAO/EAACI (World Allergy Organization/European Academy of Allergy and Clinical Immunotherapy, http://tmedihk.com/allergy- basics/). An “allergen” is an antigen which causes allergy. Most allergens reacting with IgE and IgG antibody are proteins, often with carbohydrate side chains, but in certain circumstances pure carbohydrates have been postulated to be allergens. In rare instances, low molecular weight chemicals, e.g., isocyanates and anhydrides acting as haptens, are still referred to as allergens for IgE antibodies. In the case of allergic contact dermatitis, the classical allergens are low molecular weight chemicals, e.g., chromium, nickel and formaldehyde, reacting with T cells. 

In people with atopy, rhinoconjunctivitis and rhinosinusitis, exposure to damp indoor spaces is a risk factor for the development of bronchial asthma. Rhinosinusitis associated with mold exposure doubles the risk of developing bronchial asthma (OR: 2.2; CI: 1.3–3.6) [94]. Young children with atopy appear to have an increased risk of developing bronchial asthma if there is moisture damage or mold in the bedroom or living room [95].

A numerical risk cannot be derived based on the current state of knowledge. Risk matrix 2 (Table 4 (Tab. 4)) shows a semi-quantitative risk assessment of the sensitization/allergy risk from indoor molds.

### Risk of irritanting effects

Mucous membrane irritations (MMI) of the eyes and upper respiratory tract have been described in various publications. The same applies to chronic bronchitis [1], [2].

To date, it is unclear whether individuals affected by MMI or chronic bronchitis are particularly sensitive individuals who react at lower doses, or sensitized individuals who react differently than non-sensitized individuals regardless of the dose [96]. Possible predisposing factors for MMI and chronic bronchitis can be other inflammatory processes in the area of the mucous membranes of the eyes and respiratory tract, such as infections, atopic mucosal diseases, keratoconjunctivitis sicca and dry nasal mucous membranes [29].

### Risk of toxic effects

Only molds that are potentially capable of producing toxins can be considered as triggers of toxic effects. Whether toxin formation occurs indoors in individual cases is determined by the environmental and growth conditions and, above all, the substrate [97], [98].

There are no known predisposing factors for toxic reactions caused by mycotoxins in humans. However, predispositions are conceivable at the organ level. For example, it is conceivable that a previously damaged liver (e.g., chronic hepatitis, liver cirrhosis) may be predisposed to hepatotoxic aflatoxin effects following oral ingestion of this toxin. Whether this also applies to aerogenic toxin uptake has not yet been clarified [50], [98]. A numerical risk cannot be derived based on the current state of knowledge [99].

### Risk of odor effects and mood disorders

In principle, anyone can be affected by odor effects and/or mood disorders in the event of moisture/mold damage indoors. This is a nuisance, not a health hazard.

Predisposing factors for odor effects can be genetic, age, gender and hormonal influences, imprinting, smoking, context as well as adaptation, habituation and sensitization effects [100], [101].

Predisposing factors for mood disorders can be environmental concerns, fears, conditioning and attributions, as well as a variety of diseases [39].

### Risk areas for possible health effects of mold in hospitals

The hospital can be divided into three risk areas for possible health effects of mold, which are described below.

### Low-risk area for possible health effects of mold

The low-risk area for possible health effects of mold fungi is defined in such a way that possible health risks exist at most for mood disorders, odor and/or irritation. The low-risk area corresponds to use-class II of the UBA guidelines [26]. It therefore includes all areas in the hospital that are comparable to the interiors of apartments, residential buildings, kindergartens, schools, and offices. This applies to all areas in the hospital except for areas with a medium-risk (see below) and a high-risk (see below) for possible mold health effects.

### Medium-risk area for possible health effects of mold

The medium-risk area for possible health effects of mold fungi is defined in such a way that, in addition to mood disorders, odor and/or irritation, there are also health risks for allergic reactions in atopic persons. It corresponds to use-class I of the UBA guideline [26], which is defined by special, very stringent requirements due to the individual disposition of the room users and for which the necessary measures are therefore excluded.

The medium-risk area includes:

- Areas for examining patients regarding suspected allergic diseases
- Areas of care for patients with an underlying allergic disease

### High-risk area for possible health effects of mold

The high-risk area for possible health effects of mold fungi is defined as a health risk of life-threatening allergic reactions or invasive mycoses. It corresponds to use-class I of the UBA guideline [26], which is defined by special, very stringent requirements due to the individual disposition of the room users and for which the necessary measures are therefore excluded.

The following hospital areas in which immunosuppressed patients are cared for are classified as high-risk areas:

- Intensive care units
- Areas for patients with severe respiratory tract infections
- Oncology areas, especially hemato-oncology areas
- Areas for premature babies born with deficiencies with a birth weight of less than 1,500 g
- Areas for patients with cystic fibrosis (mucoviscidosis)
- Bone marrow transplantation areas

The high-risk area also includes areas with high hygiene and infection prevention requirements, such as:

- Operating theater and areas for invasive procedures
- “Clean” areas in the central sterile supply department (CSSD)/reprocessing unit for medical devices (RUMED)
- “Clean areas” in the pharmacy

## Risk area for possible health effects of mold in medical and dental practices

Like hospitals, medical and dental practices can also be divided into three risk areas for potential mold health effects, which are described below. 

### Low-risk area for possible health effects of mold

The low-risk area for possible health effects of mold fungi is defined in such a way that possible health risks exist at most for mood disorders, odor and/or irritation. The low-risk area, which corresponds to use-class II of the UBA guidelines [26], includes practices or areas in practices that are comparable to indoor spaces in apartments, residential buildings, kindergartens, schools, and offices. This applies to all medical and dental practices and practice areas except for practices and practice areas with a medium-risk (see below) and a high-risk (see below) for possible health-related mold effects.

### Medium-risk area for possible health effects of mold

The medium-risk area for possible health effects of mold fungi is defined in such a way that, in addition to mood disorders, odor and/or irritation, there are also health risks for allergic reactions in atopic persons. It therefore corresponds to use-class I of the UBA guideline [26].

The medium-risk area includes:

- Practices and practice areas for the examination of patients regarding suspected allergic diseases
- Practices and practice areas for the care of patients with underlying allergic diseases

### High-risk area for possible health effects of mold

The high-risk area for possible health effects of mold fungi is defined in such a way that there is a health risk of life-threatening allergic reactions or mycoses. It therefore corresponds to use-class I of the UBA guidelines [26].

The high-risk area includes:

- Oncology practices and practice areas, especially hemato-oncology practices and practice areas
- Specialist practices for infectious diseases
- Practices for immunosuppressed patients who may fall under risk group 1 of the KRINKO (see Table 2 (Tab. 2))
- Practices and practice areas for patients with cystic fibrosis (mucoviscidosis)

In addition, high-risk areas also include areas in medical and dental practices with high hygiene and infection prevention requirements, such as:

- Surgical area (outpatient surgery)
- “Clean” areas of the reprocessing unit for medical devices (RUMED)

## Dealing with moisture/mold damage in hospitals and medical and dental practices depending on the risk area for possible health effects of molds

Dealing with moisture/mold damage in hospitals and medical and dental practices is based on the three risk areas described above and is described accordingly below.

### Dealing with moisture/mold damage in low-risk areas for possible health effects of mold

First of all, if not already done, the infection control staff (infection control practitioner [ICP]/“hospital hygienist”, infection control nurse [ICN] and infection control link practitioner) must be informed and involved in all decision-making processes.

In the low-risk area for health-related mold effects in hospitals and medical and dental practices, moisture/mold damage is handled in accordance with the specifications and recommendations of the UBA guidelines [26] for premises of use-class II, i.e., analogous to the handling of moisture/mold damage in apartments, residential buildings, kindergartens, schools, and offices. The remediation work must therefore be carried out by a specialized company, whereby the corresponding occupational health and safety measures must be observed [102]. For remediation control, data sheet 4–12 of the International Association of Science and Technology of Building Maintenance and Monuments Preservation (WTA) [27], which requires total spore measurements in accordance with DIN ISO 16000-20 [28] after mobilization of existing dust deposits, is used to check the success of cleaning. These clearance measurements must also be carried out by competent persons, whereby independence from the decontamination company must be ensured. The handling of affected patients and/or affected staff is carried out in accordance with the recommendations of the AWMF mold guideline “Medical clinical diagnostics for indoor mold exposure”, Update 2023 [1], [2].

### Dealing with moisture/mold damage in the medium- and high-risk areas for possible health effects of mold

In these cases, it is particularly important that the infection control staff (ICP, ICN and infection control link practitioner) are informed and involved in all decision-making processes from the outset. In addition to the procedure for moisture/mold damage in the low-risk area for health mold effects in hospitals and medical and dental practices, the following points must be implemented when dealing with moisture/mold damage in these risk areas for possible health effects of mold:

- Care of atopic patients is not permitted in these areas until they have been successfully remediated.
- Patients with bronchial asthma may not be treated in these areas until they have been successfully remediated.
- Treatment of patients with cystic fibrosis is not permitted in these areas until they have been successfully remediated.
- Care of patients with immunodeficiency/-suppression is not permitted in these areas until they have been successfully remediated.
- Care of premature babies born with deficiencies with a birth weight of less than 1,500 g is not permitted in these areas until they have been successfully remediated.
- The damaged area must be effectively sealed off from unaffected areas.
- After the remediation work, comprehensive fine cleaning must always be carried out with subsequent clearance measurements by qualified specialist companies. When treating immunodeficient patients, these so-called clearance measurement should always include the cultural determination of thermotolerant (36°C) molds and, when treating allergy patients, the mesophilic mold spectrum (25°C).
- After completion of the work, surfaces must always be disinfected and a final disinfection carried out in high-risk areas.
- The affected area may only be released by the ICP.

While mold measurements are rarely required for medical reasons, they are generally necessary if a hidden, i.e., not obviously visible, mold infestation is suspected. Only indoor air measurements can then determine whether increased exposure actually exists. Mold measurements in the indoor air and, if necessary, in building materials, are also required for planning remediation measures. They are indispensable as clearance measurements for remediation control. 

### Dealing with risk groups for possible health effects of mold in hospitals and medical and dental practices

Risk groups that require special protection from moisture/mold damage are:

- Persons with immunosuppression according to the classification of the KRINKO [45]
- Persons with severe influenza
- People with severe COVID-19
- People with cystic fibrosis (mucoviscidosis)
- Persons with bronchial asthma
- Persons with allergic bronchopulmonary aspergillosis (ABPA)
- Persons with extrinsic allergic alveolitis (EAA)

These persons must not be exposed to moisture/mold damage in any area of the hospital or practice.

### Dealing with health complaints from affected patients and/or affected staff

**Figure 4:**
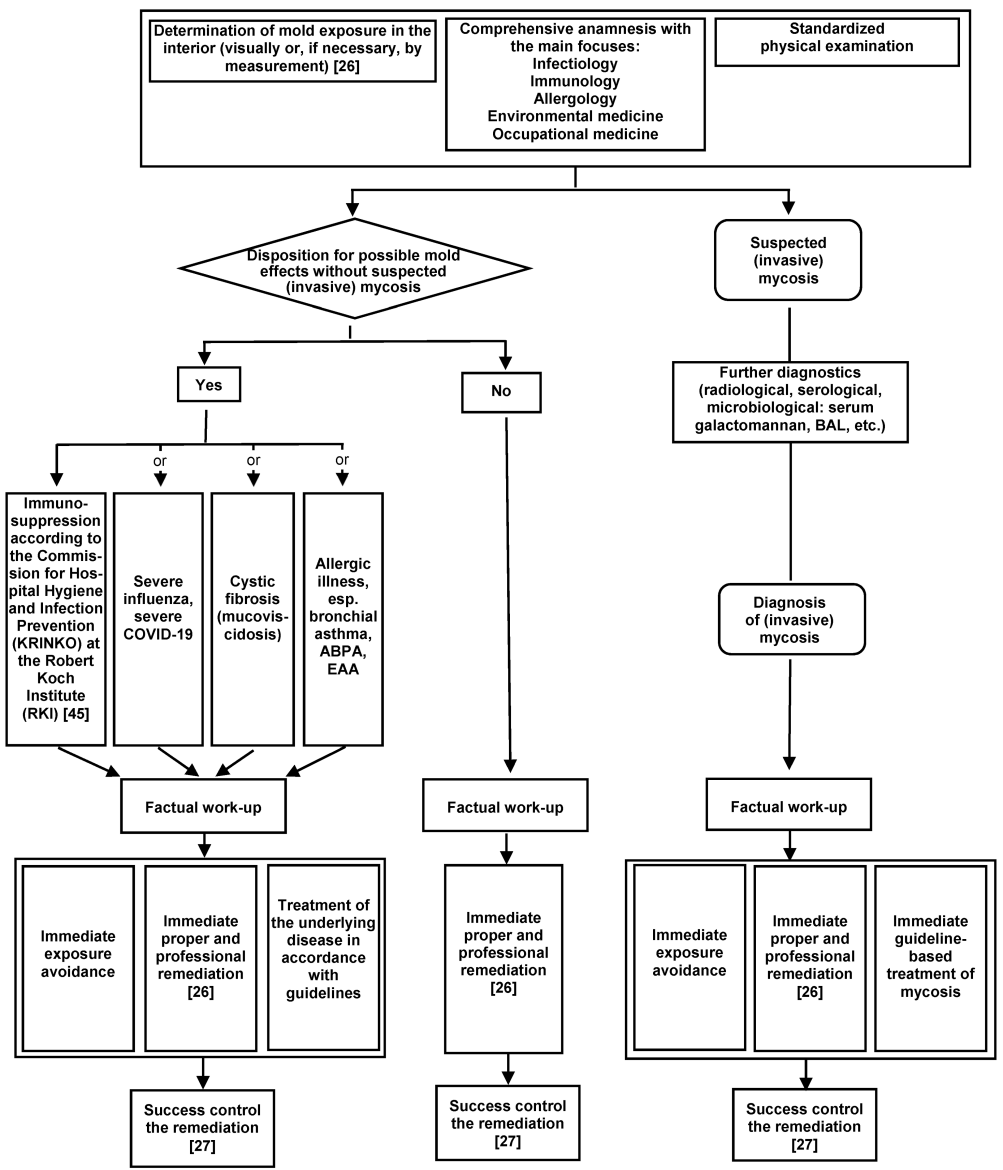
Algorithm for the clarification of suspected indoor mold-associated health disorders (BAL = bronchoalveolar lavage; ABPA = allergic bronchopulmonary aspergillosis; EAA = extrinsic allergic alveolitis; figure from [104]).

Rational diagnostics for indoor exposure to mold include a medical history and physical examination of affected patients and/or affected staff, considering risk factors, as well as conventional allergy diagnostics and, if necessary, provocation tests if allergic reactions are suspected. Sometimes cellular test systems are indicated. For invasive mold infections, please refer to the specific guidelines. Indoor mold measurements are usually not indicated for medical reasons. Only in rare cases are indoor exposure measurements recommended for species identification in connection with mold infections [103]. Indoor measurements of MVOC and/or mycotoxins as well as HBM tests for specific mold components or metabolites are not used in medical diagnostics. In case of visible mold infestation, the causes of the infestation should be clarified instead of the quantitative and qualitative determination of the mold species, so that remediation can be started quickly [1], [2]. A diagnostic algorithm is available for the procedure [104], which is shown in Figure 4 (Fig. 4).

The core messages of the AWMF mold guideline “Medical clinical diagnostics for indoor mold exposure”, Update 2023 [1], [2] are given below, which also contain the core recommendations of the guideline. The strength of the recommendation is expressed by the following terms: strong recommendation: “shall”, recommendation: “should”, open recommendation: “can be considered”. The strength of consensus was determined according to AWMF guidelines: >95%. = strong consensus, >75% to ≤95% = consensus, >50% to ≤75% = majority agreement, ≤50%= no majority agreement:

1. Mold infestation to a relevant extent should not be tolerated indoors for precautionary reasons. To assess the extent of damage, please refer to the “Guidelines for the prevention, detection and remediation of mold infestation in buildings” published by the Federal Environment Agency [26]*. Modified 2023, consensus strength >95%*
2. The most important measures in case of indoor mold infestation are to identify the cause and carry out proper remediation [26]. *Checked 2023, consensus strength >95%*
3. For medical indication, indoor mold measurements are rarely useful. As a rule, both a quantitative and a qualitative determination of mold species can be dispensed with in case of visible mold infestation. Rather, the causes of the infestation should be clarified, and then the infestation and primary causes should be eliminated. *Modified 2023, consensus strength >95%*
4. In medical diagnostics for mold exposure, environmental monitoring of mycotoxins in indoor air and house dust has no indication. *New 2023, consensus strength >95%*
5. In medical diagnostics for mold exposure, environmental monitoring of microbial volatile organic compounds (MVOC) in indoor air has no indication. *New 2023, consensus strength >95%*
6. Exposure to mold can generally lead to irritation of the mucous membranes (mucous membrane irritation (MMI)), odor effects and mood disorders. *Checked 2023, consensus strength >95%*
7. Specific clinical pictures associated with mold exposure relate to allergies and mold infections (mycoses). *Checked 2023, consensus strength >95%*
8. Physicians shall, in cases of a suspected association between indoor moisture/mold damage and conditions for which there is no evidence of such an association (e.g., acute idiopathic pulmonary hemorrhage in children, arthritis, autoimmune diseases, chronic fatigue syndrome (CFS), endocrinopathies, gastrointestinal effects, cancers, airborne mycotoxicoses, multiple chemical sensitivity (MCS), multiple sclerosis, neuropsychological effects, neurotoxic effects, sudden infant death syndrome, renal effects, reproductive disorders, rheumatism, thyroid disorders, sick building syndrome (SBS), teratogenicity, and urticaria), inform affected individuals objectively about the current state of knowledge. *Modified 2023, consensus strength >95%*
9. Risk groups requiring special protection are:
  a) Persons under immunosuppression according to the classification of the Commission for Hospital Hygiene and Infection Prevention (KRINKO) at the RKI [45]
  b) Persons with severe influenza
  c) Persons with severe COVID-19
  d) Persons with cystic fibrosis (CF; mucoviscidosis)
  e) Persons with bronchial asthma
*Modified 2023, consensus strength >95%*
10. Individuals who are allergic to mold and those with diseases that weaken the immunological defense system shall be informed about the dangers of mold exposure indoors and about measures to prevent and minimize such exposure. *Modified 2023, consensus strength >95%*
11. In principle, a large number of mold species can cause sensitization and allergies in case of corresponding exposure. Compared to other environmental allergens, however, the allergenic potential is to be regarded as lower overall [90], [91]. *Modified 2023, consensus strength >95%*
12. As polysensitized individuals, atopic patients often also have IgE antibodies against molds, although this does not necessarily mean that they are ill. The clinical severity of the allergic reaction does not correlate with the level of the specific IgE titer. *Modified 2023, consensus strength >95%*
13. The core elements of a type I allergy diagnosis are medical history, skin prick test, determination of specific IgE antibodies, and provocation testing. In case of allergic bronchopulmonary aspergillosis (ABPA), the determination of specific IgG antibodies should also be performed. In the case of extrinsic allergic alveolitis (EAA), only the determination of specific IgG antibodies shall be performed serologically. *Modified 2023, consensus strength >95%*
14. The detection of specific IgE or a positive reaction in the skin test initially only mean that a specific sensitization to corresponding allergens is present. A clinically relevant allergy only becomes apparent in connection with typical allergic symptoms. *Modified 2023, consensus strength >95%*
15. A negative result of a skin test or a specific IgE test for molds does not reliably exclude sensitization to molds. The reasons for this include the varying composition and quality of test extracts or the absence of relevant allergens. *Modified 2023, consensus strength >95%*
16. The determination of specific IgG antibodies in connection with the diagnosis of an immediate-type mold allergy (type I allergy) has no diagnostic significance and shall therefore not be performed. This also applies to the detection of immune complexes, e.g., using the Ouchterlony test. *Modified 2023, consensus strength >95%*
17. Galactomannan in serum shall only be performed for the diagnosis of suspected invasive pulmonary aspergillosis, otherwise there is no indication in the diagnosis of mold exposure. *New 2023, consensus >95%.*
18. The determination of eosinophil cationic protein (ECP) and β-1,3-D-glucan (BDG) in serum has no indication and shall not be performed in medical diagnostics in case of mold exposure. *New 2023, consensus strength >95%*
19. The basophil degranulation test and histamine release (histamine liberation test (HLT), the basophil activation test (BAT) using flow cytometry and the determination of other mediators (sulfidoleukotriene release test, cellular antigen stimulation test (CAST-ELISA) are used in special diagnostics, but should not be performed in basic allergy diagnostics. *New 2023, consensus strength >95%*
20. Lymphocyte transformation tests (LTT) for molds are not indicated as a diagnostic procedure [105] and shall therefore not be performed. *Modified 2023; consensus strength >95%*
21. The whole blood test is not a suitable instrument for detecting mold sensitization and shall therefore not be performed. *New 2023, consensus strength >95%*
22. Invasive mold infections are rare and are most likely to occur by inhalation. In practice, of the molds classified in risk groups 2 and 3 according to TRBA 460 [53]. *Aspergillus fumigatus* is the most important mycosis pathogen. Individuals with general strong or very strong immune deficiency (according to KRINKO grade 2 and 3 [45]) are predominantly affected. In case of a corresponding disposition, this risk shall be given special attention. *Modified 2023, consensus strength >95%*
23. Microbiological, immunological, molecular biological and radiological methods are core elements of mold infection diagnostics and shall be used depending on the indication. *Modified 2023, consensus strength >95%*
24. Human biomonitoring of mycotoxins has no indication in medical diagnostics for indoor mold exposure, and shall therefore not be performed. *New 2023, consensus strength >95%*
25. The following diagnostic methods shall not be used for indoor mold exposure because there is insufficient scientific evidence (without claim of completeness): Detection of molds in the blood, determination of IgA antibodies directed against molds, determination of lymphocyte subpopulations, determination of cytokines, determination of oxidative stress, visual contrast sensitivity test (VCS test), tear film break-up time. *New 2023, consensus strength >95%*
26. The following diagnostic methods shall not be used for indoor mold exposure due to a lack of medical and scientific evidence (list is non-exhaustive): electroacupuncture according to Voll, bioresonance procedures, pendulum, Vega test, decoder dermography, biotonometry, biotensor, Kirlian photography (plasma print procedure, energetic terminal point diagnosis), regulation thermography according to Rost, auriculodiagnostics, kinesiology, aurascopy, iris diagnostics, cytotoxic blood tests, provocation and neutralization test (PN test). *New 2023, consensus >95%*

An abridged version [106] of the of the AWMF mold guideline “Medical clinical diagnostics for indoor mold exposure”, Update 2023 [1], [2] in generally understandable language for those affected by moisture/mold damage indoors can be made available to affected patients and/or affected staff.

### Mold outbreaks in health care settings

The Centers for Disease Control and Prevention (CDC) provides a guideline for healthcare-associated mold outbreaks [107].

### Prevention of moisture and mold in health care settings

Lamphier (2023) [108] and the CDC [107] give the following advice for controlling mold in health care settings:

- Control humidity levels
- Promptly identify and correct leaks
- Reduce dust during construction
- Place immunocompromised patients in positive pressure rooms to reduce the risk of mold infections
- Use other strategies for mold mitigation, e.g., air filtration, thorough cleaning and drying after flooding, and ventilating areas such as showers, laundry, and cooking areas

## Conclusions

If mold/moisture damage occurs in a hospital or practice, the fastest possible elimination of the causes and remediation of the damage have the highest priority. The hygiene specialists must be involved in this. The infection control practitioner (ICP) makes all relevant decisions. For medical reasons, mold measurements in the affected rooms are generally not indicated. For patients with an increased health risk, such indoor measurements carry the risk of a potentially critical prolonged exposure. Indoor mold measurements should not be carried out on people in these risk groups as a precautionary measure to rule out mold growth if there is no evidence of it (except possibly for the purpose of clearance measurements). Rather, all measures that help to prevent moisture damage are crucial for prevention.

Mold measurements are only required if a hidden, i.e., not obviously visible, mold infestation is suspected, for the planning and control (clearance measurement) of remediation measures and, in rare cases, for species determination in connection with mold infections.

## Cross-references

**Mold guide from the Federal Environment Agency:** Indoor Air Hygiene Commission of the Federal Environment Agency. Guidelines for the prevention, detection and remediation of mold infestation in buildings. Dessau: Federal Environment Agency; 2024 [last accessed on 2025 Jan 29]. Available from: https://www.umweltbundesamt.de/sites/default/files/medien/479/publikationen/240513_uba_fb_schimmelleitfaden_0.pdf

**WTA data sheet on mold damage remediation 4–2:** Objectives and control of mold damage remediation indoors. Berlin: DIN Media GmbH; 2021 [last accessed on 2025 Jan 29]. Available from: https://www.dinmedia.de/de/technische-regel/wta-merkblatt-4-12/342998074

**AWMF mold guideline update 2023:** Hurraß J, Heinzow B, Walser-Reichenbach S, Aurbach U, Becker S, Bellmann R, Bergmann KC, Cornely OA, Engelhart S, Fischer G, Gabrio T, Herr CEW, Joest M, Karagiannidis C, Klimek L, Köberle M, Kolk A, Lichtnecker H, Lob-Corzilius T, Mülleneisen N, Nowak D, RabE U, Raulf M, Steinmann J, Steiß JO, Stemler J, Umpfenbach U, Valtanen K, Werchan B, Willinger B, Wiesmüller GA. AWMF mold guideline “Medical clinical diagnostics for indoor mold exposure” – Update 2023 AWMF Register No. 161/001. Allergol Select. 2024;8:90-198; DOI: 10.5414/ALX02444E

**AWMF mold guideline update 2023 — short version for those affected:**

Hurraß J, Heinzow B, Walser-Reichenbach S, Aurbach U, Becker S, Bellmann R, Bergmann KC, Cornely OA, Engelhart S, Fischer G, Gabrio T, Herr CEW, Joest M, Karagiannidis C, Klimek L, Köberle M, Kolk A, Lichtnecker H, Lob-Corzilius T, Mülleneisen N, Nowak D, Rabe U, Raulf M, Steinmann J, Steiß JO, Stemler J, Umpfenbach U, Valtanen K, Werchan B, Willinger B, Wiesmüller GA. AWMF-Schimmelpilz-Leitlinie „Medizinisch klinische Diagnostik bei Schimmelpilzexposition in Innenräumen" – Update 2023. Stand 05.09.2023. AWMF-Register-Nr. 161/001 (https://register.awmf.org/de/leitlinien/detail/ 161-001) – Kurzfassung für Betroffene mit einem Feuchte-/Schimmelschaden im Innenraum – (Stand 05.09.2023) [last accessed on 2025 Jan 29]. Available from: https://register.awmf.org/assets/guidelines/161- 001k_S2k_Medizinisch-klinische-Diagnostik-bei-Schimmelpilzexposition-in-Innenraeumen_2024-03.pdf

**Diagnostic algorithm for indoor mold exposure:** Hurraß J, Nowak D, Heinzow B, Joest M, Stemler J, Wiesmüller GA. Indoor mold – Important considerations for medical advice to patients. Dtsch Ärztebl Int 2024; 121. DOI: 10.3238/arztebl.m2024.0018

## Notes

### Competing interests

The authors declare that they have no competing interests.

## Figures and Tables

**Table 1:**
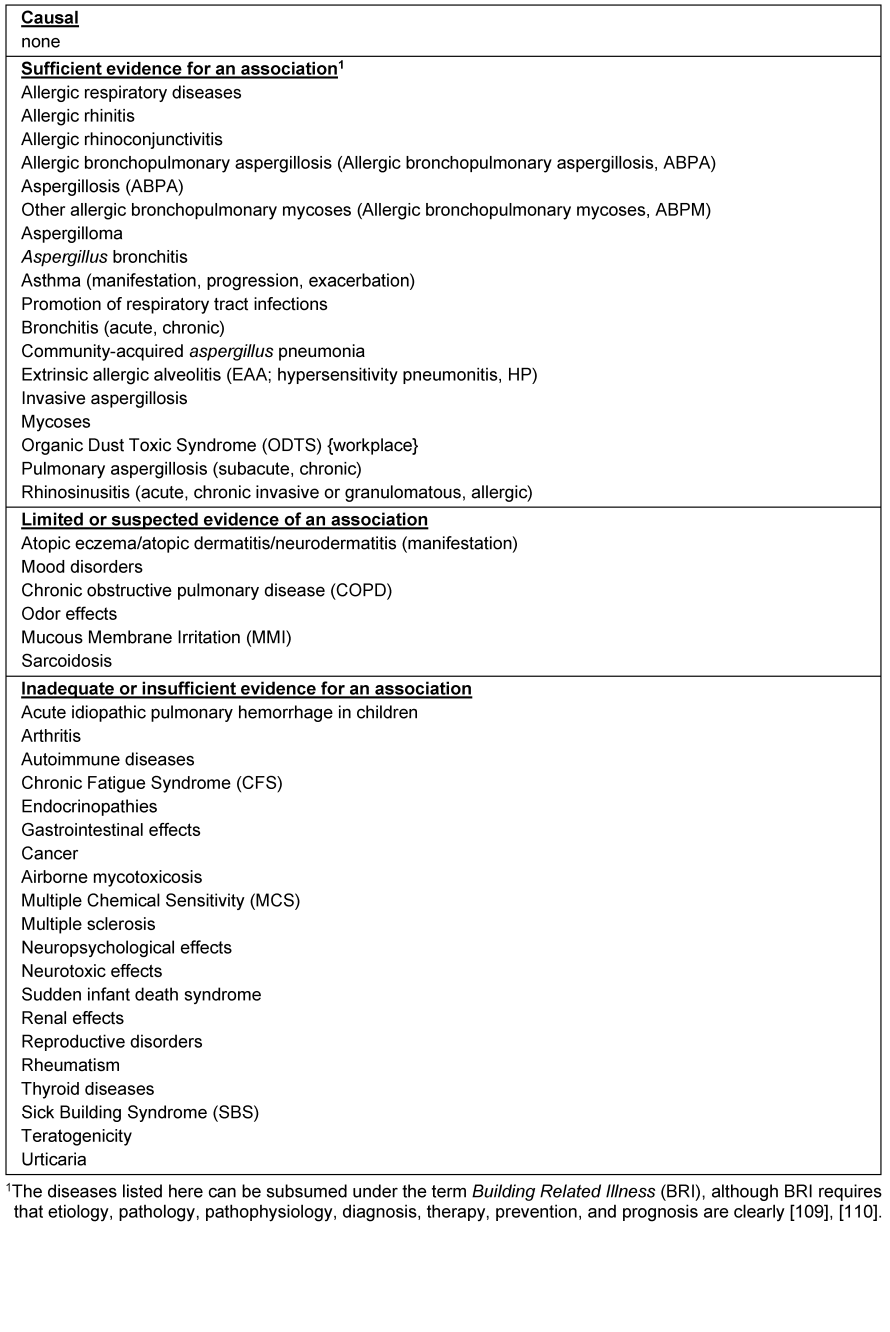
Evidence for the relationship between indoor damp/mold exposure and disease (in alphabetical order) [1], [2].

**Table 2:**
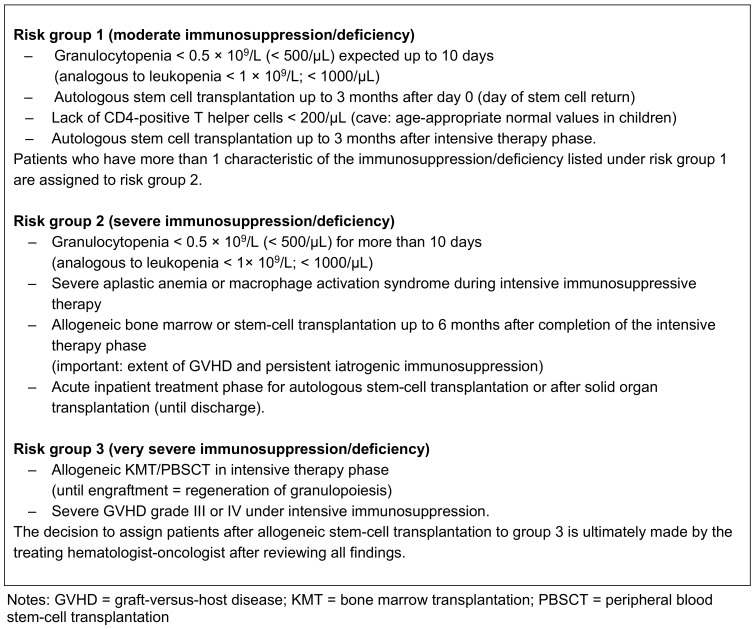
Immunosuppression risk groups of the Commission for Hospital Hygiene and Infection Prevention (KRINKO) at the Robert Koch Institute Berlin [45].

**Table 3:**
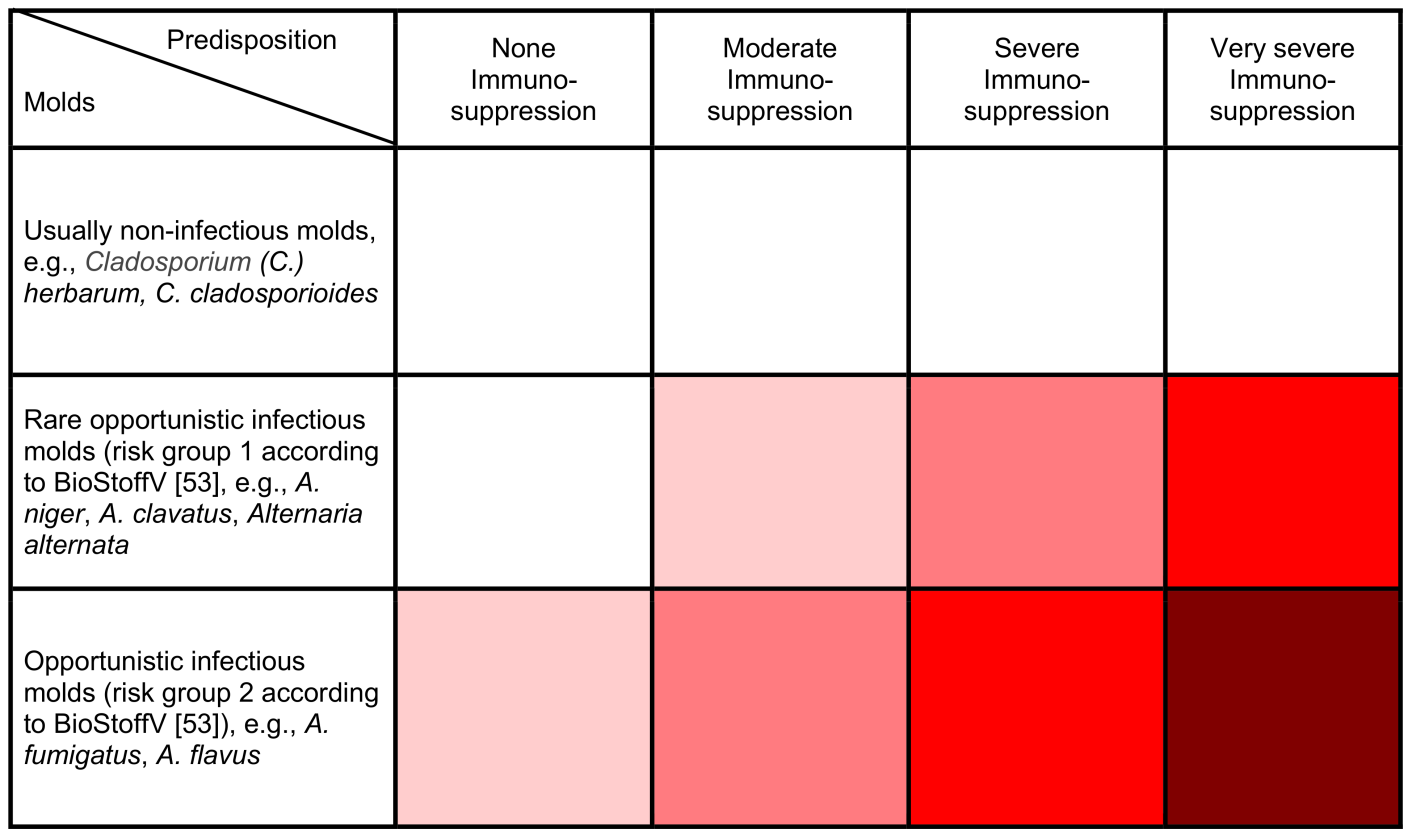
Risk matrix 1 – risk of infection from mold (the darker the box, the greater the potential health risk)

**Table 4:**
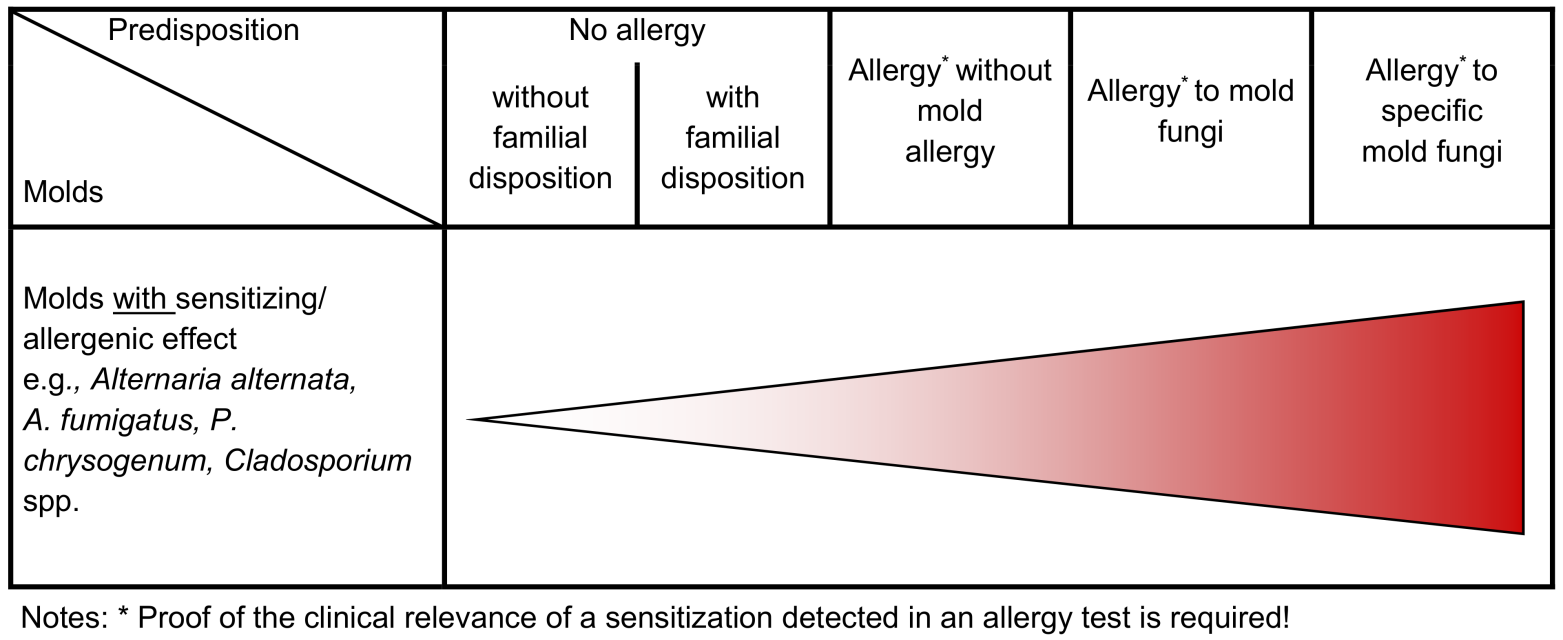
Risk matrix 2 – sensitization/allergy risk due to molds (the darker the color, the greater the potential health risk)
